# Supplementary material for: The nucleosome DNA entry-exit site is important for transcription termination and prevention of pervasive transcription
Source: eLife. 2020 Aug 26;9:e57757. doi: 10.7554/eLife.57757 (PMC7449698; doi:10.7554/eLife.57757)
Supplement: Supplementary file 1. [file elife-57757-supp1.docx]

**Supplementary File 1. Oligonucleotides**

| *SNR47-YDR042C* F | GGCGGTAACGTAAATCAGAGTAGC | Northern probe |
| --- | --- | --- |
| *SNR47-YDR042C* R | GAGACCTAGTCGTTTGTTAGCTG | Northern probe |
| *SNR48-ERG25* F | CCTTGGCGCAGAAGACTTTCTCTTC | Northern probe |
| *SNR48-ERG25* R | GCATACACAGGCGTACGCATACAAG | Northern probe |
| *SNR13-TRS31* F | CGTAGCGCTGCATATATAATGCG | Northern probe |
| *SNR13-TRS31* R | GATGCAGAAGTCGCTGTGCTGGAG | Northern probe |
| *SNR48* F | GCCTTTTTCTTGAATTGTCAATCCGCCC | Northern probe |
| *SNR48* R | GGATGTGAAGTTTAAGTACTCTCCATTCAATGAATAC | Northern probe |
| *SCR1* F | CAACTTAGCCAGGACATCCA | Northern probe |
| *SCR1* R | AGAGAGACGGATTCCTCACG | Northern probe |
| *SNR48-*SB F | TTACTATGATTAAACAGACCGAGGGAGAA | ChIP-qPCR |
| *SNR48-*SB R | AACGGGTTTAAACGGCTGCC | ChIP-qPCR |
| NotI-SB | ATAAGAATGCGGCCGCTACCGCGTGCTAAGGCGCCTTA | Superbinder amplification from TOPO-TA + SB |
| SB-NotI | TAAACTATGCGGCCGCGTATACGCCTTACACGCGCCTAGA | Superbinder amplification from TOPO-TA + SB |
| EcoRI-SB | CCGGAATTCTACCGCGTGCTAAGGCGCCTTA | Superbinder amplification from TOPO-TA + SB |
| SB-XhoI | CCAGCTCGAGGTATACGCCTTACACGCGCCTAGA | Superbinder amplification from TOPO-TA + SB |
| *URA3* R | CCGCCTGCTTCAAACCGCTAACA | Superbinder integration |
| *URA3* F | TGCAGTACTCTGCGGGTGTATAC | Superbinder integration |
| *SNR48*-SB F | AGGGCCTACGTAGAAGATGATGTAAAAGTAGATTCGCATCTACCGCGTGCTAAGGCGCCT | Superbinder integration |
| *SNR48*-SB R | TGACTACACCGGGTAACGAATGGATTGCGTAATATACCGAGTATACGCCTTACACGCGCC | Superbinder integration |
