# Supplementary material for: The nucleosome DNA entry-exit site is important for transcription termination and prevention of pervasive transcription
Source: eLife. 2020 Aug 26;9:e57757. doi: 10.7554/eLife.57757 (PMC7449698; doi:10.7554/eLife.57757)
Supplement: Supplementary file 2. [file elife-57757-supp2.docx]

**Supplementary File 2. Yeast strains**

| **Strain** | ***MAT*** | **Genotype** |
| --- | --- | --- |
| KY319  (FY111)* | **a** | *spt6-140 his4-912∂ lys2-128∂ ura3-52 trp1∆63* |
| KY325  (FY896) | **a** | *spt10∆::TRP1 his4-912∂ lys2-128∂ leu2∆1 ura3-52 trp1∆63 suc2∆uas(-1900/-390)* |
| KY812  (FY1991) | **a** | *(hht1-hhf1)∆::LEU2 (hht2-hhf2)∆::KanMX his3∆200 lys2-128∂ leu2∆1 ura3-52 trp1∆63* [pDM9 *= URA3/CEN/ARS/HHT1-HHF1*] |
| KY943  (FY406) | **a** | (*hta1-htb1*)Δ::*LEU2* (*hta2-htb2)Δ::TRP1 his3Δ200 lys2-128δ leu2Δ1 ura3-52* [pSAB6 = *URA3/CEN/ARS/HTA1-HTB1*] |
| KY1437 | **a** | *rtt109∆::KanMX his3∆200 leu2∆0 ura3-52* |
| KY3219 | α | *leu2∆1::ADH1p-HIS3-CYC1::LEU2 (hht1-hhf1)∆::LEU2 (hht2-hhf2)∆::KanMX his3∆200 lys2-128∂ leu2∆1 ura3-52 trp1∆63* [pDM9 *= URA3/CEN/ARS/HHT1-HHF1*] |
| KY3220 | α | *leu2∆1::ADH1p-SNR47(70)-HIS3-CYC1::LEU2 (hht1-hhf1)∆::LEU2 (hht2-hhf2)∆::KanMX his3∆200 lys2-128∂ leu2∆1 ura3-52 trp1∆63* [pDM9 *= URA3/CEN/ARS/HHT1-HHF1*] |
| KY3221 | **a** | *SNR48-SB (hht1-hhf1)∆::LEU2 (hht2-hhf2)∆::KanMX his3∆200 lys2-128∂ leu2∆1 ura3-52 trp1∆63* [pDM9 *= URA3/CEN/ARS/HHT1-HHF1*] |
| KY3232 | α | *RPB3-3xFLAG::KanMX KanMX::GAL1p-YLR454W HA-SPT15 (hht1-hhf1)∆::LEU2 (hht2-hhf2)∆::KanMX his3∆200 lys2-128∂ leu2∆1 ura3-52 trp1∆63* [pDM9 *= URA3/CEN/ARS/HHT1-HHF1*] |
| KY3354 | α | *his3∆200 lys2-128∂ leu2∆1 ura3-52 trp1∆63 (hht1-hhf1)∆::LEU2 (hht2-hhf2)∆::KanMX snf2∆::HIS3* [pDM9 *= URA3/CEN/ARS/HHT1-HHF1*] |
| KY3502 | **a** | *his4-912∂ lys2-128∂ leu2∆1 ura3-52 trp1∆63 (hht1-hhf1)∆::LEU2 (hht2-hhf2)∆::KanMX* [pDM9 *= URA3/CEN/ARS/HHT1-HHF1*] |
| KY3503 | α | *his3∆200 lys2-128∂ leu2∆1 ura3-52 trp1∆63 suc2∆uas(-1900/-390) (hht1-hhf1)∆::LEU2 (hht2-hhf2)∆::KanMX* [pDM9 *= URA3/CEN/ARS/HHT1-HHF1*] |
| KY3506 | α | *his3∆200 lys2-128∂ leu2∆1 ura3-52 trp1∆63 KanMX::GAL1p-FLO8-HIS3 (hht1-hhf1)∆::LEU2 (hht2-hhf2)∆::KanMX* [pDM9 *= URA3/CEN/ARS/HHT1-HHF1*] |
| KY3511 | α | *leu2∆1 ura3-52 trp1∆63 HHT1-HA::KanMX (hht2-hhf2)∆::KanMX* |
| KY3575 | α | *his3∆200 lys2-128∂ leu2∆1 ura3-52 trp1∆63 set2∆::HIS3 (hht1-hhf1)∆::LEU2 (hht2-hhf2)∆::KanMX* [pDM9 *= URA3/CEN/ARS/HHT1-HHF1*] |

*FY strains were provided by Fred Winston.
